# Tuberculosis and COVID-19 in the elderly: factors driving a higher burden of disease

**DOI:** 10.3389/fimmu.2023.1250198

**Published:** 2023-09-27

**Authors:** Anna Allué-Guardia, Jordi B. Torrelles, Alex Sigal

**Affiliations:** ^1^ Population Health Program, Texas Biomedical Research Institute, San Antonio, TX, United States; ^2^ International Center for the Advancement of Research and Education (I•CARE), Texas Biomedical Research Institute, San Antonio, TX, United States; ^3^ Africa Health Research Institute, Durban, South Africa; ^4^ Centre for the AIDS Programme of Research in South Africa, Durban, South Africa; ^5^ School of Laboratory Medicine and Medical Sciences, University of KwaZulu-Natal, Durban, South Africa

**Keywords:** SARS-CoV-2, *Mycobacterium tuberculosis*, COVID-19, TB, elderly, immunity, infectious diseases

## Abstract

*Mycobacterium tuberculosis* (*M.tb*) and SARS-CoV-2 are both infections that can lead to severe disease in the lower lung. However, these two infections are caused by very different pathogens (*Mycobacterium vs.* virus), they have different mechanisms of pathogenesis and immune response, and differ in how long the infection lasts. Despite the differences, SARS-CoV-2 and *M.tb* share a common feature, which is also frequently observed in other respiratory infections: the burden of disease in the elderly is greater. Here, we discuss possible reasons for the higher burden in older adults, including the effect of co-morbidities, deterioration of the lung environment, auto-immunity, and a reduced antibody response. While the answer is likely to be multifactorial, understanding the main drivers across different infections may allow us to design broader interventions that increase the health-span of older people.

## Introduction

The older adult population (> 60 years old) is projected to double to 2 billion by 2050 (1, 2). Natural lung aging is associated with progressive changes at both the cellular and organ level, including cellular senescence and chronic inflammation among others (3). This causes a decline in lung function and impaired immunological responses (4–7), which would be expected to influence the response to respiratory infections.

Coronavirus disease 2019 (COVID-19) and tuberculosis (TB) are both predominantly respiratory diseases but do not have a great deal in common beyond that. COVID-19 results from severe acute respiratory syndrome coronavirus 2 (SARS-CoV-2) infection, a virus that in most people persists for a few weeks or less and is cleared by the adaptive immune response. Protection against symptomatic SARS-CoV-2 infection correlates strongly with the levels of neutralizing antibodies against the virus (8). In support of this, the Omicron variant of SARS-CoV-2 was able to extensively re-infect people with pre-existing immunity (9) because it had high-level escape from neutralizing antibodies elicited by previous infection or vaccination (10). In contrast, TB, caused by *Mycobacterium tuberculosis* (*M.tb*), can persist indefinitely in the infected individual (11, 12). Further, TB generally follows a bimodal age pattern, with higher risk of severe disease in children below 5 years of age and adult individuals of > 30 years old (13, 14), while severe COVID-19 is more common in older adults and pediatric COVID-19 deaths are relatively rare (15). These differences may be due to the fact that the immune responses in TB and COVID-19 are different.

Despite the differences, these two infections share common features: first, both are strongly affected by immunosuppression (e.g. during HIV infection), indicating that their control strongly depends on T cell and/or antibody responses, which are compromised by the CD4 T cell depletion and dysregulation during HIV infection (16–24). Indeed, TB is one of the cardinal diseases leading to the death of people living with HIV (PLWH) in the pre-ART era (25, 26). On the other hand, the most striking effect of HIV co-infection in COVID-19 happens in advanced HIV disease (defined as a CD4 T cell counts of less than 200 cells per microliter), where prolonged SARS-CoV-2 infection can last for months (27–33), leading to extensive SARS-CoV-2 genome evolution. A second common feature, which will be the focus of this review, is the remarkably higher disease burden in the elderly population (34). This is also true for most respiratory infections such as respiratory syncytial virus (35, 36), influenza (37, 38), and even rhinovirus, which is usually a mild upper respiratory tract infection, but can become a more severe lower respiratory infection in the elderly, very young children, or immunocompromised people (39).

Globally, COVID-19 has a mortality rate of about 1% (40), although this is influenced and fluctuates depending on many factors, including phenotypic and genotypic host factors, host immunity, and SARS-CoV-2 variants, among others. The elderly are at a higher risk of having more severe disease, which manifests as a lower respiratory tract infection that may require hospitalization, intensive care, and ventilation. It also results in higher mortality (41–43). The increase in the probability to die from COVID-19 as a function of age is dramatic: relative to the under-55 age group, mortality increases 8-fold in the 55-64 age group and 62-fold in the over 65 age group (43).

In contrast to the 1% mortality rate from COVID-19, mortality from TB disease is roughly a quarter of the TB incidence (44). That is, about a quarter of people diagnosed with TB disease will die. However, most people who are exposed to *M.tb* do not progress to symptomatic disease and instead have subclinical or asymptomatic infection for years. In this case, the infection is controlled by the host immune response (45, 46). In the elderly population, such subclinical or asymptomatic infection has a higher chance to develop into TB disease (47–49). Indeed, more than 90% of TB cases in older individuals result from reactivation of latent TB infection (LTBI) (50). Elderly people that develop TB disease have high mortality, mainly due to treatment failure. A recent report evaluating data from four countries shows that the treatment success rate among people with TB < 65 years old is 82% but decreases among the older age groups to 76% in 65−74 year-olds, 65% in 75−84 year-olds, and 46% in ≥85 year-olds (51).

**Figure 1:**
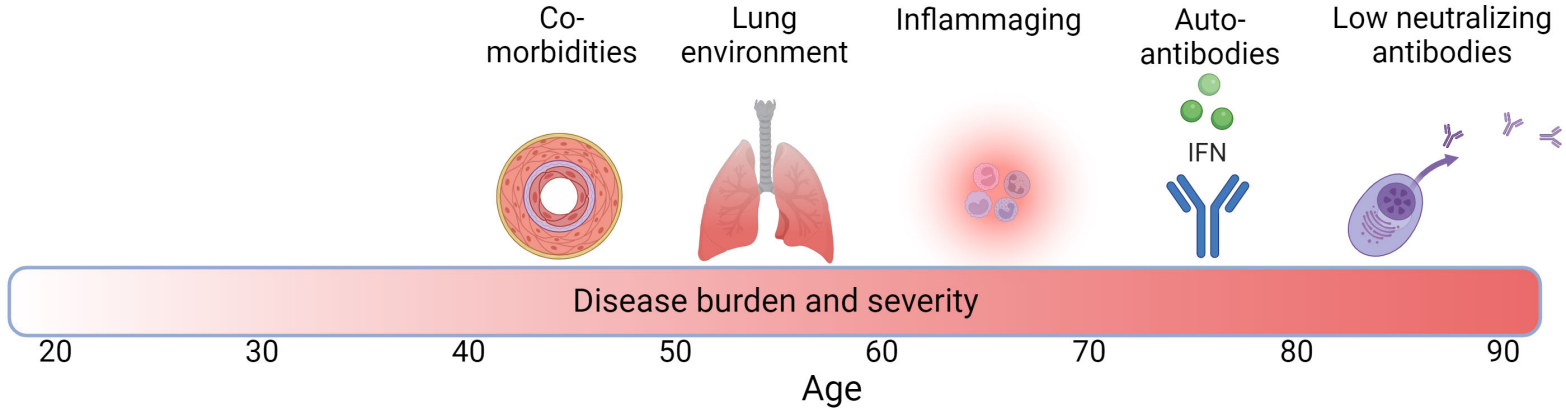
Factors associated with increased TB and COVID-19 disease burden with age across the life span. Darker red in bar denotes higher disease burden and severity and numbers denote age. Created with BioRender.com.

There are multiple factors that may interact with each other and potentially play a role in the higher disease burden in COVID-19 and TB in the elderly, and their contribution may differ between the two infections. These include age-associated inflammation (inflammaging), a less effective immune response due to immunosenescense, and a highly oxidized lung environment (**Figure 1**). Although observed less frequently, other factors such as an age-related increase in autoantibodies (autoimmunity) may play a role in higher COVID-19 severity in the elderly. In addition, increasing numbers of people living with comorbidities in the elderly population may be particularly important. These factors tend to arise at different times along the life span (**Figure 1**). In the next sections, we outline examples for each of these factors, including how they may exacerbate COVID-19 and TB in older individuals.

## Inflammaging and immunosenescence

The process of aging is associated with a decline in immune functions marked by immunosenescence, resulting in increased susceptibility to autoimmunity, malignancies, and infectious diseases (4, 5, 52–58). Immunosenescence is relatively well characterized in the adaptive immune system. Age-related adaptive immune dysfunction is related to a lingering level of low-grade inflammation, immune dysfunction, increased number of memory T cells, and the loss ability of T cells to respond to antigens, as well as irreversible T cell loss of proliferation capacity. Interestingly, viral (e.g. SARS-CoV-2) and bacterial (e.g. *M.tb*) infections can also increase the extent of immune senescence, adding to the increased immune dysfunction and inflammation, especially in the elderly population (reviewed in detail in (59)).

Senescence in the innate immune system, where innate immunity is the first response to infection, is less well-characterized. Evidence for macrophage senescence during aging is supported by decreased pro-inflammatory responses of human (60–64) and mouse phagocytes to lipopolysaccharide (LPS) stimulation (65–70), which could be linked to age-related alterations in Toll-Like Receptor (TLR) expression and/or signaling which recognizes pathogen-associated molecular patterns (65, 66, 71–73).

Still, cellular immunosenescence does not fully explain increased circulating pro-inflammatory cytokines seen in elderly people, non-human primates, and old mice (61, 74–76), or the increased expression of pro-inflammatory genes with aging in several organs (77–81). This has led to a second paradigm, termed inflammaging, in which chronic, low-grade inflammation develops with increasing age in tissues that are frequently exposed to innate immune stimulation and oxidative stress (82, 83). Inflammaging occurs in the human lung, with increased numbers of macrophages and neutrophils in the lung alveolar lining fluid (ALF) of elderly individuals, as well as increased levels of IL-6 and IL-8 (84, 85). Specifically, IL-6 is the commonly used biomarker of inflammaging (86). There is also increased p38 MAPK phosphorylation and nuclear localization of NF-κB (87–89), a critical regulator of inflammation. Resident alveolar macrophages are more activated in the elderly (90–92) and have increased production of pro-inflammatory cytokines in response to TLR stimulation (93). Taken together, there is strong evidence that chronic inflammation occurs in the lungs as we age.

How inflammaging affects *M.tb* and SARS-CoV-2 infection is not completely understood. However, in both infections, a balanced immune response is thought to be critical both for infection control and to prevent immune system mediated damage. Tumor necrosis factor (TNF), the upstream activator of the NF-κB system, is elevated in inflammaging. High levels of TNF lead to reduced control of *M.tb* through programmed cell necrosis of activated macrophages via the mitochondrial-lysosomal-endoplasmic reticulum signaling circuit (94–96). Since macrophages are the primary host cells of *M.tb* as well as the most important line of defense against this pathogen, macrophage death in turn increases *M.tb* replication since the bacilli are able to robustly grow in the dead infected cells (97).

Immunosenescence and inflammaging are also suspected to contribute to severe COVID-19 in the elderly as well as to persistence of symptoms following acute disease (98, 99). Severe SARS-CoV-2 infection is characterized by a cytokine storm that, combined with a dysfunctional immune response in the elderly, leads to the accumulation of immune cells in the lungs and overproduction of pro-inflammatory molecules such as IL-6 (a marker of inflammaging), resulting in more tissue damage (100–102). High levels of pro-inflammatory molecules (hyperinflammatory syndrome) promote the survival of neutrophils via decreased apoptosis (103); and persistent increased systemic levels of neutrophils and monocytes in COVID-19 patients are associated with increased disease severity (104). Age-associated dysregulation and senescence of T-cells may also influence the immune response to SARS-CoV-2 (105). As seen in HIV infection, CD4 T cell depletion and dysregulation may lead to the inability to clear SARS-CoV-2 infection, most likely due to the inability to generate antibodies which will effectively neutralize the virus (27). This would be expected since CD4 T cells are critical to facilitate the antibody response to infection (106). Lastly, SARS-CoV-2 infection might also increase chronic inflammation in the elderly, resulting in a higher chance of long-term sequelae even after viral clearance (long-COVID) (107). New therapies targeting age-associated pathways may be critical to reduce COVID-19 mortality and/or long-term sequelae in the aging population (108).

## Lung environment in the elderly in the context of TB and COVID-19

Local inflammation and oxidation occur in the aging lung and influences the ALF (109, 110). ALF is a surfactant which reduces surface tension and allows the lung alveoli to expand. It also functions in multiple ways in the innate immune response to lung pathogens. Here we will focus on the ALF as an example of how the lung environment can change with age and its impact on TB and COVID-19.

ALF is generated, secreted, and recycled by alveolar epithelial type II cells (ATII), and is essential for maintaining lung homeostasis (111, 112). ALF in elderly individuals degrades quickly and is not regenerated efficiently because of ATII senescence. In addition, low-grade chronic inflammation in old age is expected to alter ALF component production and activity, due in part to biochemical modifications because of the alveolar oxidation state. We and others have shown that components of human ALF including collectins (which bind pathogen surface oligosaccharides or lipids and mark the pathogen for the innate immune response), surfactant protein (SP)-A and SP-D, homeostatic hydrolytic activities (hydrolases), surfactant lipids, and the complement system are critical elements of the innate immune system during *M.tb* infection (113–115) and play important roles in *M.tb*-phagocyte encounters (116–119). Indeed, SP-A upregulates the expression of the mannose receptor in macrophages, which in turn favors *M.tb* survival within phagocytes. In contrast, SP-D can directly bind *M.tb* clustering bacteria, favoring recognition and uptake by phagocytes driving better control of the infection (110).

In a study defining the molecular composition of ALF in the aging lung, our findings demonstrate that pro-inflammatory cytokines are increased, SP-A and SP-D and complement components are significantly increased but dysfunctional, ALF hydrolases are decreased, and surfactant lipids are oxidized in both mice and humans (109). Further, a recent quantitative proteomic profiling of the lung environment of human adult *vs.* elderly ALF investigated molecular fingerprints, pathways, and regulatory networks that characterize the alveolar space in old age compared to younger individuals (120). ALF from elderly individuals had significantly increased production of matrix metalloproteinases, markers of cellular senescence, antimicrobials, and proteins of neutrophilic granule origin, among others, suggesting that neutrophils could be potential contributors to the dysregulated alveolar environment with increasing age.

Consistent with reduced ALF functionality with age, *M.tb* exposed to human ALF obtained from older adults showed increased intracellular growth in macrophages and ATIIs (121–123), as well as increased bacterial burden and lung tissue damage in mice (121). In addition, *M.tb* exposed to ALF from healthy 18- to 45-year-old adults upregulated key cell envelope genes associated with amino acid, carbohydrate, and lipid metabolism, as well as genes associated with redox homeostasis and transcriptional regulators, while *M.tb* exposed to ALF from 60+ year-old individuals showed lower transcriptional responses (124). The changes in ALF in aging support the concept that the pulmonary environment can modify mucosal immune responses, thereby increasing the susceptibility to pulmonary infections in the elderly population.

How ALF influences SARS-CoV-2 infection is mostly unknown. However, patients with severe COVID-19 sometimes harbor IgA autoantibodies against pulmonary SP-B and SP-C, blocking the function of the lung surfactant lipid layer and potentially contributing to alveolar collapse and poor oxygenation (125). Other studies indicate that levels of SP-D in blood could be used as a biomarker for COVID-19 severity as a result of the impairment of the pulmonary barrier caused by prolonged inflammation (126). Still, how the levels, status, and function of ALF components in the alveolar environment determine the outcome of *M.tb* and SARS-CoV-2 infection and disease severity of TB and COVID-19, respectively, still needs to be elucidated in detail.

## Reduced adaptive immune responses

The adaptive immune response is essential to control both *M.tb* and SARS-CoV-2. CD4 T cells are critical in the orchestration of both the antibody and cellular adaptive immune response to infections (106, 127, 128). For SARS-CoV-2, the strongest correlate of protection against symptomatic infection is the level of pre-existing neutralizing antibody immunity (8, 129). Thus, SARS-CoV-2 neutralizing antibody levels are studied extensively as a function of age. A complication of measuring neutralizing antibody levels after infection is that higher disease severity elicits higher antibody levels (130). However, it is possible to distinguish between neutralizing antibody production capacity and disease severity by measuring neutralizing antibody levels to SARS-CoV-2 post-vaccination, with much of the data coming from mRNA vaccines.

One of the first studies examining the neutralizing antibody response to the Pfizer BNT162b2 mRNA vaccine against SARS-CoV-2 found that the fraction of people with a detectable neutralizing antibody response decreased slowly as a function of age up to the age of 80, with almost all individuals responding to the vaccine. After 80, the probability to elicit a neutralizing antibody response plummeted and was close to zero at 90, although the number of individuals in this part of the age range was small in the study (131). A second study used a cutoff of 55 years for who is elderly and showed substantially lower neutralizing antibody levels in the older age group after the first dose of an mRNA vaccine (132). However, this difference decreased with the second dose. A third study done in Singapore also found that people over 60 had lower neutralizing responses with an mRNA vaccine. However, they showed a strong increase in neutralizing antibodies with a third, booster dose (133). The benefit of a booster dose was recapitulated in a group of over-80-year-olds who did not have an antibody response to the first two doses (134).

The role of neutralizing antibodies in the immune response to *M.tb* is currently unclear. However, the reduced ability to mount an effective neutralizing antibody response may indicate an overall less effective adaptive immune response in the elderly, which would reduce *M.tb* control.

## Autoimmunity

An essential component of the initial immune response to both *M.tb* and SARS-CoV-2 is interferon (IFN), which orchestrates the innate immune response to infection. In TB, the type II interferon IFN-γ activates macrophages and enables them to initiate maturation and acidification of the *M.tb*-containing phagosome, as well as other antimicrobial responses (135). The failure of this process to kill the internalized bacilli leads to macrophage death and *M.tb* growth in the dead infected cells (97). Mice deficient in IFN-γ quickly succumb to TB (136, 137).

The role of type I interferons during *M.tb* infection is not completely understood, with some studies reporting a host protective role *vs.* other studies suggesting a detrimental role under different host-*M.tb* encounter settings (138, 139). However, type I interferons including IFN-α are an important component of the innate immune response to SARS-CoV-2 (100), and they are rapidly induced in early stages of the infection (140). Multiple SARS-CoV-2 genes attempt to interfere with IFN (141–144). Individuals with inborn errors in type I IFN immunity are much more prone to severe COVID-19 (145) and mice deficient for type I IFN have reduced activation of CD4 and CD8 T cells and reduced recruitment of monocytes and monocyte-derived macrophages to the lung (146).

Anti-IFN antibodies might block IFN binding to IFN receptors, impairing its antiviral effect (147). There have been sporadic case reports of anti-IFN antibodies increasing susceptibility to mycobacterial infections (148–150) or shown to be elevated at the site of infection in advanced TB patients (151). In contrast, SARS-CoV-2 infection is reported to be more severe in individuals with autoantibodies to type I IFN. In one study, 101 of 987 patients with severe COVID-19 have been found to have these autoantibodies, while none of the 663 individuals with asymptomatic or mild SARS-CoV-2 infection had anti-IFN type I antibodies (152). The prevalence of anti-IFN type I antibodies was found to be strongly age dependent (153, 154). Autoantibodies neutralizing high concentrations of IFN-α were present in 0.18% of individuals between 18 and 69 years, 1.1% of individuals between 70 and 79 years, and 3.4% of people >80 years of age (154). Autoantibodies are unlikely to completely explain the higher susceptibility of the elderly population to severe COVID-19. However, such autoimmunity may be a contributing factor in a subset of people (155) and an example of an age dependent affect which is highly variable between people of the same age. Also, antibodies to other host proteins are known to increase with age (156). This may potentially add to disease pathology in a similar way.

## Comorbidities

An important aspect of the shift towards a global aging population is increasing chronic illness. The top 10 comorbidities associated with the elderly population include hypertension (58%), high cholesterol (47%), arthritis (31%), ischemic heart disease (29%), diabetes (27%), chronic kidney disease (18%), heart failure (14%), depression, Alzheimer disease and dementia (11%) and chronic obstructive pulmonary disease (COPD, 11%) (157). Some of these comorbidities overlap with known risk factors for TB (diabetes) and higher COVID-19 severity (obesity, hypertension, high cholesterol, and diabetes). Thus, a common risk factor for TB and COVID-19 which increases in prevalence in the elderly is diabetes. This is not surprising, as diabetes leads to higher mortality from a range of infectious diseases (158).

Projections suggest that the global incidence of diabetes will double in the next 20 years, with 40% of this estimated to result from the aging population (159, 160). The elderly are at high risk for developing type 2 diabetes due to underlying insulin resistance, impaired pancreatic function, and a higher obesity prevalence linked to changes in body composition and physical inactivity (161, 162). The resulting high blood sugar can cause serious complications such as heart disease, kidney problems, and loss of vision. Furthermore, diabetes in older adults is associated with a higher risk for chronic microvascular and cardiovascular complications and common geriatric syndromes and is linked to higher mortality (163). People with diabetes have altered cytokine release by macrophages and T cells, impaired neutrophil recruitment, and decreased levels of type I interferons as well as reduced numbers of new populations of dendritic cells (DCs) and natural killer (NK) cells (164). Also, the diabetic lung is characterized by structural modifications such as abnormalities in small vessels (alveolar diabetic microangiopathy or microvascular disease) (165), as well as alterations in the interstitial environment (166, 167) and autonomic neuropathy with loss of autonomic innervation in bronchioles (168), which might contribute to adverse outcomes in respiratory diseases (169).

While the interactions between TB and diabetes in the elderly are not completely understood, people with diabetes are 3 times more likely to develop pulmonary TB, especially those with poorly controlled diabetes (170, 171). *In vitro* and *in vivo* studies have found reduced association and uptake of *M.tb* by monocytes from people with diabetes and alveolar macrophages from mice with chronic diabetes, as well as reduced innate immune responses and a persistent systemic hyper-inflammation in TB-diabetic individuals (172–174). Diabetes also promotes TB reactivation due to impaired T cell immunity, specifically because of decreased IFN-γ production by CD4 T cells (175). In addition, cavitary disease (where cavities are abnormal, thick-walled, air-filled spaces in the lung which result when a granuloma encasing *M.tb* liquifies and ruptures) is more frequently observed in elderly TB patients with diabetes than in non-diabetic elderly patients, suggesting that diabetes promotes cavitation in the aging lung parenchyma (176).

In addition, TB might pose a risk of developing diabetes (177). A persistent inflammatory state in response to TB disease might result in secondary metabolic effects such as “stress hyperglycemia”, defined as temporary hyperglycemia caused by stress during acute illness (178). It has been suggested that stress hyperglycemia may negatively influence TB treatment outcomes, although this relationship is still poorly understood (178).

Individuals with diabetes are at a higher risk for SARS-CoV-2 severe disease and mortality (34, 179–185). According to an analysis done in the South African population, the hazard ratios for mortality range from 3 to 12 for ≥20 years old public-sector patients, with the mortality risk increasing as blood sugar control decreases. Risk may be lower in other populations, perhaps due to better diabetes control: about 3-fold higher for mortality as reported in a meta-analysis (181). While the worse disease outcome of SARS-CoV-2 infection in diabetics is well established, diabetics are not necessarily at higher risk of infection with SARS-CoV-2 (179), indicating that not all aspects of immunity are equally compromised.

## *M.tb* and SARS-CoV-2 co-infection

Respiratory infections tend to interact in one of two ways. They can synergize, with the cardinal example being *Streptococcus pneumoniae* bacterial infection after influenza virus infection. This happens because the virus causes damage to the mucosal surface, allowing the bacteria to attach better and invade more easily (186). In addition, the type I interferon response to the virus decreases phagocyte function and therefore control of the bacteria by phagocytosis (186). The other possible interaction is antagonism, and usually happens between viruses. This is called super-infection exclusion and occurs because the type I interferon antiviral response trigged by one virus can inhibit other viruses (187). Two studies by independent groups examined experimental SARS-CoV-2/*M.tb* co-infection in K18-hACE2 transgenic mice. Both groups found that SARS-CoV-2 infection did not affect *M.tb* loads or associated pathology. They also observed that *M.tb* infected mice were more resistant to SARS-CoV-2 infection (188, 189). This is consistent with a report that intravenous administration of BCG, a live attenuated TB vaccine developed from *Mycobacterium bovis*, protects mice against lethal SARS-CoV-2 challenge (190). Thus, there is currently no mechanistic basis for synergy between SARS-CoV-2 and *M.tb.* There is still a poor understanding of the pathology and immunological changes associated with *M.tb*/SARS-CoV-2 co-infection (191), as recently reviewed in (192).

In terms of epidemiology, some studies suggest that the dysregulated immunity during *M.tb* infection is associated with increased susceptibility and severity of COVID-19 and *vice versa* (193–198). There is also some evidence suggesting that in the elderly population, TB and COVID-19 may be associated with increased mortality compared to each disease occurring alone (199–201). Mechanisms may include increased lung damage in TB patients with COVID-19, resulting in impaired lung function (202) or higher risk of TB reactivation after COVID-19 infection due to depletion of CD4 T cells and excessive lung fibrosis. Worse outcomes of co-infection may also be because of shared clinical, immunological, and social determinants (203–206), as well as compromised linkage to care for HIV and TB in a pandemic environment (207). In our own South Africa based cohort of SARS-CoV-2 infected individuals, we did not observe a clear enrichment of active TB disease (208) relative to the observed incidence in the South African population (209).

## Conclusions and future perspectives

Aging has a negative effect on the outcomes of both SARS-CoV-2 and *M.tb* infection, and may be considered a subtype of immunosuppression/dysregulation which varies widely between individuals of a similar age. This may be because the effect is multi-factorial and involves age-related inflammation (inflammaging) and senescence of immune cell subsets, as reviewed previously (210). It is particularly damaging to the adaptive arm of the immune response which is critical to control both infections. In addition to that, the lung environment itself also changes with age, and many of the changes are associated with the reduced ability of alveolar fluid to perform its innate immune functions. Aging also increases autoimmunity, and in a subset of individuals this may manifest as autoantibodies to immune mediators such as interferons, with the result that innate immunity becomes less effective at reducing pathogen replication. This is an example of how the effects of aging can be heterogeneous. Lastly, co-morbidities such as type II diabetes increase with age, and such co-morbidities, though they do not necessarily increase the chances of infection, are risk factors for more severe disease if infection does occur.

Conversely, SARS-CoV-2 and *M.tb* infections may accelerate age-related processes. For example, *M.tb* infection and TB treatment, as well as long-COVID, might result in cardiovascular complications and induce cardiovascular disease (211), an important comorbidity associated with the older population. Also, SARS-CoV-2 is associated with increased oxidative stress, which also plays a role in the pathogenesis of diabetes (212).

Some of the processes described here are already targets for interventions. For example, the elderly are prioritized for COVID-19 vaccination to compensate for the less effective immune response to SARS-CoV-2 (213). Other interventions, for example better control of diabetes, are available but are not uniformly implemented due to health systems challenges, particularly in low- and middle-income countries (214). Interventions which may increase lung health at a given stage of life are yet little explored, but have the potential to work across pathogens to decrease the effects of infection, which could translate to substantial gains in the health span of aging populations.

## Author contributions

All authors listed have made a substantial, direct, and intellectual contribution to the work and approved it for publication.
